# Withdrawal of inhaled corticosteroids from patients with COPD with mild or moderate airflow limitation in primary care: a feasibility randomised trial

**DOI:** 10.1136/bmjresp-2022-001311

**Published:** 2022-08-30

**Authors:** Timothy H Harries, Gill Gilworth, Christopher J Corrigan, Patrick Murphy, Nicholas Hart, Mike Thomas, Patrick T White

**Affiliations:** 1 School of Population Health and Environmental Sciences, King’s College London, London, UK; 2 Department of Asthma Allergy and Respiratory Science, King's College London, London, UK; 3 Lane Fox Respiratory Unit, Guy's and St Thomas' NHS Foundation Trust, London, UK; 4 PCPS, University of Southampton, Southampton, UK

**Keywords:** COPD Pharmacology, COPD epidemiology

## Abstract

**Background:**

Inhaled corticosteroids (ICS) are frequently prescribed outside guidelines to patients with chronic obstructive pulmonary disease (COPD) with mild/moderate airflow limitation and low exacerbation risk. This primary care trial explored the feasibility of identifying patients with mild/moderate COPD taking ICS, and the acceptability of ICS withdrawal.

**Methods:**

Open feasibility trial. Outcome measures included prevalence of suitable participants, feasibility of their identification, their willingness-to-accept open randomisation to ICS withdrawal or continuation over 6 months follow-up.

**Results:**

392 (13%) of 2967 patients with COPD from 20 practices (209 618 population) identified as eligible for ICS withdrawal by electronic search algorithm. After individual patient record review, 243 (62%) were excluded because of: severe airflow limitation (65, 17%); one or more severe or two or more moderate COPD exacerbations in the previous year (86, 22%); asthma (15, 4%); and severe comorbidities (77, 20%). After exclusion, 149 patients with COPD were invited to participate and 61 agreed to randomisation. At clinical assessment, 10 patients exhibited undocumented airflow reversibility (forced expiratory volume in 1 s (FEV_1_) reversibility >12% and >200 mL); 2 had suffered two or more undocumented, moderate exacerbations in the previous year; 7 had severe airflow limitation; and 2 had normal spirometry. Finally, 40 were randomised. One patient died and one was lost to follow-up. 18 (45%) of the 38 (10 withdrawal and 8 usual care) exhibited previously undocumented FEV_1_ variability suggestive of asthma, supported in the withdrawal group by significant associations with elevated fractional exhaled nitric oxide (p=0.04), elevated symptom score (p=0.04), poorer quality of life (p=0.04) and atopic status (p=0.01).

**Conclusions:**

Identifying primary care patients with mild/moderate COPD suitable for ICS withdrawal is feasible but requires real-time verification because of unreliable recording of exacerbations and lung function. Suitable patients accepted randomisation to ICS withdrawal or continuation for the purposes of future studies. Follow-up compliance was high. Nearly 50% of participants with a diagnosis of mild/moderate COPD demonstrated previously undocumented FEV_1_ variability during follow-up, mandating monitoring for at least 6 months following withdrawal to exclude undiagnosed asthma.

**WHAT IS ALREADY KNOWN ON THIS TOPIC**

- Inhaled corticosteroids (ICS) are prescribed frequently outside guidelines to patients with mild or moderate chronic obstructive pulmonary disease (COPD).
- Little is known of the feasibility and acceptability to these patients of ICS withdrawal.

**WHAT THIS STUDY ADDS**

- Identification of patients with mild or moderate COPD suitable for ICS withdrawal in primary care is feasible and the process of withdrawal is acceptable. A high proportion of patients have forced expiratory volume in 1 s variability.

**HOW THIS STUDY MIGHT AFFECT RESEARCH, PRACTICE OR POLICY**

- Evidence for future definitive randomised controlled trials of the withdrawal of ICS among patients with COPD with mild or moderate disease is provided. Withdrawal of ICS should prompt extended monitoring to exclude undiagnosed asthma.

## Background

Therapy with higher-dosage inhaled corticosteroids (ICS) in combination with long-acting bronchodilators (LABA) reduces the risk of chronic obstructive pulmonary disease (COPD) exacerbations in patients with severe or very severe airflow limitation (forced expiratory volume in 1 s (FEV_1_) <60% predicted).^1^ This treatment is at the cost of increased risk of pneumonia and fractures,^2 3^ which add to the financial burden for health services.^4^ Many patients with a diagnosis of COPD and FEV_1_>60% predicted are also prescribed these medications^5–7^ despite lack of evidence of benefit in randomised controlled trials.^8^ Uncertainty about the effectiveness of ICS therapy in reducing the risk of COPD exacerbations has led to interest in the impact of withdrawing this medication across the spectrum of COPD severity.^9 10^ Withdrawal of ICS therapy in primary care from those patients with COPD unlikely to benefit is acceptable in principle.^11^

International guidelines fail to clarify matters; according to the current Global Initiative for Chronic Obstructive Lung Disease (GOLD) report,^12^ ICS therapy may be withdrawn from any patient with COPD with persistent dyspnoea or exacerbations if ‘the original indication for ICS was inappropriate’, or if there are ‘adverse effects’, or if there has been a ‘lack of response’. Neither the magnitude of, nor the time scale over which these effects should be assessed are clearly defined. The European Respiratory Society (ERS) provide a conditional recommendation for ICS withdrawal, following exclusion of asthma, in those patients in whom each of the following criteria are fulfilled: an absence of a history of a severe acute exacerbation of COPD (AECOPD); fewer than two moderate exacerbations in a year; a baseline blood eosinophil count <300 cells/µL.^13^ The ERS guidelines do not specify the year in which these exacerbations are noted. The positive or negative predictive values of the eosinophil threshold of <300 cells/µL on a single occasion, whether or not a patient is using ICS therapy, have not been established.^14^ In both guidelines the indications for withdrawal are based on authoritative consensus arising from evidence of association in post-hoc analyses of uneven quality.^15^

Although withdrawal of ICS therapy from patients with a true diagnosis of COPD suffering from mild or moderate airflow limitation might confer net clinical benefit to the patients and financial benefit to the health service, these patients may be difficult to differentiate from others with coexisting asthma or a missed diagnosis of asthma, in whom withdrawal of ICS therapy is clearly contraindicated.^16 17^ Eosinophilic COPD, based on blood eosinophil count, proposed as evident in 10–15% of the total patient with COPD population,^17–19^ may reflect coexisting COPD and asthma. These patients manifest greater bronchodilator reversibility, a better response to ICS therapy, elevated fractional exhaled nitric oxide (FeNO) and are more likely to have a history of asthma and/or atopy.^17 20^ Among patients with COPD with a high frequency of exacerbations, an elevated blood eosinophil count has been associated with an improved response to ICS therapy in randomised controlled trials, although the quality of the evidence, derived from post-hoc analyses and observational studies, is inconsistent.^15 21^ In the current study we examined the feasibility and accuracy of identifying patients with true COPD and mild or moderate airflow limitation and low exacerbation history from existing general practice health records, the acceptability of randomisation to withdrawal of ICS or continuation of ICS to these patients and finally the feasibility of conducting open, randomised controlled trials of ICS withdrawal from these patients.

## Methods

This was a study of the feasibility of identifying patients with COPD and mild or moderate airflow limitation suitable for, and willing to participate in, an open, randomised controlled clinical trial of withdrawal of ICS therapy. Set in primary care in the UK, the trial was conducted between 2017 and 2019. Analysis conformed to the Consolidated Standards of Reporting Trials guidelines for pilot and feasibility trials.^22 23^ We have previously reported a qualitative study of the acceptability of the intervention and a study of the method of identifying and recruiting participants.^11 24^

### Patient and public involvement

Patients and the public were first involved during the planning and application process of the feasibility trial. A patient advisory group, drawn from the local British Lung Foundation Breathe Easy Group, was convened. This group provided advice to the project team on the writing of all patient literature and communication tools, on the development of patient data collection instruments, on the recruitment and retention of participants in the project and on the burden of the proposed investigations. A patient representative also joined the trial steering group. A key element of the trial was the assessment of participants’ views of the invitation to be randomised to withdraw higher dosage ICS or to continue using them. Members of the patient advisory group gave us the opportunity to address areas of sensitivity in this trial in which the possible responses of patients were largely unknown. They reviewed participant information sheets and recommended greater emphasis on the adverse effects of taking higher dosage ICS. These priorities informed the development of participant information resources.

### Study population, recruitment and randomisation

London (UK) general practices were recruited for the feasibility trial. From a previous study in London,^25^ it was anticipated that recruitment of a population of patients sufficient to conduct the study would require screening of the patients in a minimum of 10 practices. Participating practices conducted an algorithm-based digital search (online supplemental figure S1) of their electronic patient records for possible eligible patients. Subsequent individual searches of these patients’ records to confirm likely eligibility were required to confirm: a recorded diagnosis of COPD; no recorded history or diagnosis of asthma; FEV_1_≥50% predicted; fewer than two moderate COPD exacerbations and no history of a severe exacerbation (hospital admission) in the previous year; and regular consumption of higher-dosage ICS therapy (>400 µg/day beclometasone dipropionate or equivalent). This dosage was chosen to reflect both the positive effect on exacerbation frequency reduction and the increased risk of adverse effects from ICS above this threshold.^8 26^ Likely eligible patients were invited to a clinical review at their own practice. The review was conducted by a general practitioner (GP) member of the research team (THH) appointed as an honorary clinician in the general practice. The research GP conducted assessments of participants and managed prescribing decisions related to the research. Patients were provided with written information in advance and advised that, if suitable, they would be invited to take part in the feasibility trial. At clinical review: patients’ histories were assessed; confirmation made that no inhaled LABA and long-acting muscarinic antagonist (LAMA) therapies had been used in the 12 hours beforehand; spirometry including surveillance for bronchodilator reversibility (FEV_1_ reversibility >12% and >200 mL) after maximal possible bronchodilatation using 400 µg salbutamol delivered via a spacer device was carried out^27^; counselling about adherence to treatment was provided; and inhaler technique was checked.

10.1136/bmjresp-2022-001311.supp1 Supplementary data

Participant inclusion criteria were: age ≥45 years; recorded COPD diagnosis; FEV_1_/forced vital capacity (FEV_1_/FVC) <0.7; FEV_1_≥50% predicted; current user of ICS therapy >400 µg/day beclometasone dipropionate or equivalent, alone or in combination with LABA therapy, for at least the previous 3 months; no history of asthma; no bronchodilator reversibility (defined as FEV_1_ reversibility >12% and >200 mL) on testing; fewer than two moderate COPD exacerbations and no history of a severe exacerbation in the previous year^28^; and ICS prescription outside the recommendations of the GOLD report.^29^ Exclusion criteria were: active lung cancer; breathlessness due to heart disease; current severe mental illness including depression, psychosis, anxiety; current alcohol or drug dependence; continuous oral steroid use; and deemed unsuitable by their GP. Participants provided informed consent. Randomisation with minimisation by age, gender and FEV_1_ was carried out to test the acceptability of: randomisation to potential participants; allocation to withdrawal of ICS or usual care; and retention in the study. Randomisation was carried out independently by King’s Clinical Trials Unit. All baseline and follow-up assessments were carried out by THH. Allocation was not concealed at assessment because the outcome of the study was feasibility and acceptability. Precise estimates for hypothesis testing were not made as the study was designed as a feasibility trial.

## Intervention

Participants allocated to the intervention arm were instructed about ICS withdrawal while continuing all other current COPD treatment. Control arm participants were instructed to continue all current COPD treatment. Withdrawal of ICS therapy followed the London Borough of Lambeth Clinical Commissioning Group’s step-down inhaler guide for COPD (online supplemental figure S2).^30^ Participants in the ICS withdrawal group were telephoned at 2, 4 and 6 weeks after baseline to check on the progress of withdrawal and to assess and record symptoms or events of concern. Each was invited for assessment at their GP’s surgery at 3 and 6 months after baseline. The baseline measures, except for bronchodilator reversibility testing, were repeated at each assessment. Reports of disease exacerbations were verified by checking the electronic patient records. A moderate exacerbation of COPD was defined as treatment with antibiotics and/or oral corticosteroids in the community, and a severe exacerbation was defined by admission to hospital.^31^

### Outcome measures and assessment intervals

At baseline, participants completed a questionnaire of demographic and medical details including age, sex, smoking status and pack-year history, evidence of clinical type 1 allergy (hay fever, seasonal or perennial rhinitis, eczema, past severe allergic reaction or specific allergies), current medication and exacerbation frequency in the previous year. At baseline, 3 and 6 months, the following assessments were carried out: spirometry according to the American Thoracic Society (ATS)/ERS guidance^32^; COPD-specific quality of life with the Chronic Obstructive Pulmonary Disease Assessment Test (CAT)^33^ and the Chronic Respiratory Questionnaire Self-Administered Standardised (CRQ-SAS).^34^ A CAT score of ≥10 points indicates high symptom burden.^35^ The minimum clinically important difference (MCID) in CAT is a score change of ≥2.^33 36^ The CRQ-SAS consists of four domain scores: dyspnoea, fatigue, emotional functioning and mastery.^34^ The MCID for the CRQ-SAS is a change of ≥0.5 per domain. Participants also completed the Hospital Anxiety and Depression Scale. A score of ≥11 in either domain suggests the presence of anxiety or depression.^37^

Blood was taken for eosinophil count (absolute cells/mL), and periostin concentration (ng/mL). Samples were transported within 2 hours to the local National Health Service hospital pathology laboratory. Eosinophil analysis was carried out within 6 hours. Samples for periostin were taken into biochemistry tubes, centrifuged and stored at −28°C for batch analysis following the 6-month assessment. FeNO levels were assessed with a Circassia NIOX VERO hand-held electrochemical analyser, as recommended by the ERS.^38^ Participants were seated, without nose clip, advised to inhale to their total lung capacity through the mouthpiece, then exhale, maintaining an appropriate and constant expiratory flow rate by following an animated interface on the analyser.^38^ A FeNO level <25 ppb was considered normal and ≥25 ppb was considered elevated as recommended by the ATS.^39^ Each FeNO assessment was repeated three times and a mean reading recorded.

## Statistical analysis

The sample size for the feasibility trial was based on an estimation of the prevalence of patients who would deem it acceptable to be randomised to withdraw or continue their ICS therapy. We assumed that 90% of patients who had expressed interest in participating would find randomisation acceptable. At least 75 patients would have given 95% confidence of being within ±7% of the true figure of acceptability if the proportion who found randomisation acceptable was 90%. Data were analysed using SPSS V.27 (IBM, Chicago, USA). Conformity of the continuous/parametric outcome measures with a parametric distribution was verified using the Shapiro-Wilk normality test. Parametrically distributed data are presented as the mean±SD, and skewed data as the median and IQR. Group comparisons between ICS withdrawal and usual care groups of normally distributed data were analysed using the independent samples t-test. For skewed data, group comparisons were calculated using the Mann-Whitney U test. Categorical variables were compared using the χ^2^ test.

## Results

**Figure 1:**
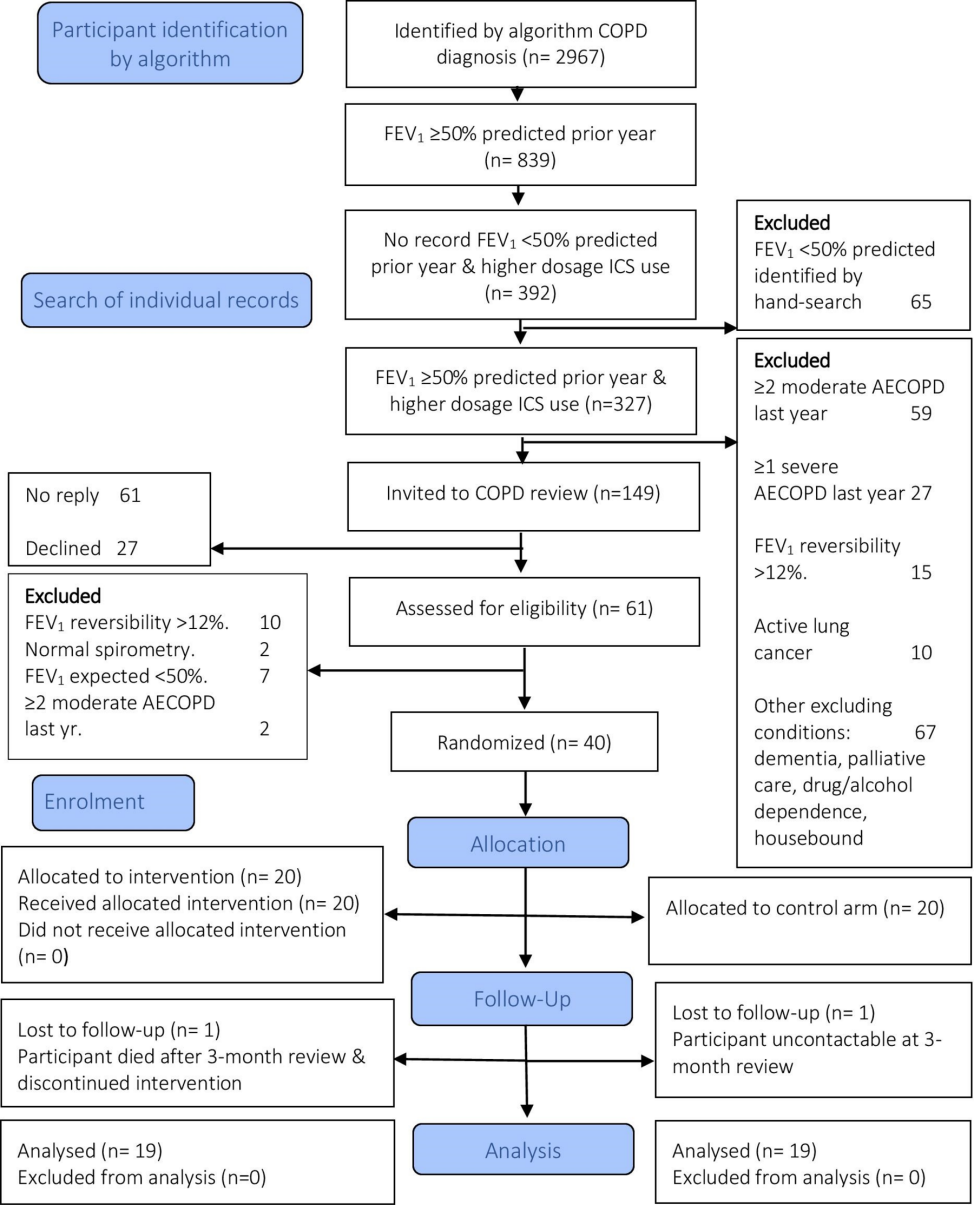
Feasibility trial Consolidated Standards of Reporting Trials flow diagram with recruitment flow. AECOPD, acute exacerbation of COPD; COPD, chronic obstructive pulmonary disease; FEV_1_, forced expiratory volume in 1 s; ICS, inhaled corticosteroids.

Twenty general practices, with a total patient population of 209 618, took part. A summary of COPD characteristics identified by digital search algorithm in participating practices is shown in table 1. Records of the 392 (13.2%) patients with COPD identified by the digital search algorithm were reviewed individually (figure 1). Inconsistencies in diagnosis and exacerbation recording were common.^40^ Repeat prescriptions of antibiotics and prednisolone (rescue packs) were often provided without associated evidence of an exacerbation. Two hundred and forty-three (62%) of the 392 patients identified by the algorithm were ineligible for the trial (figure 1). One hundred and forty-nine eligible patients were invited for clinical review.^24^ Sixty-one patients attended the review. Twenty-one patients were excluded because of characteristics identified at the review that had not been found in their electronic patient records. Ten patients exhibited evidence suggestive of asthma (FEV_1_ reversibility >12% and 200 mL), two had suffered two or more moderate COPD exacerbations in the prior year, seven had severe airflow limitation and two had normal spirometry. Forty patients were eligible and agreed to be randomised to withdrawal of ICS therapy or continuation of usual care. None of these 40 participants demonstrated FEV_1_ reversibility defined as >12% and >200 mL in the Global Initiative for Asthma (GINA) guidelines of 2021,^27^ following bronchodilatation. One patient died during the study of an unrelated cause. One patient defaulted from further follow-up after 3 months.

**Table 1:** COPD characteristics of patients in participant practices

Practice characteristics	Participant practices (n=20)
Population (practice mean)	209 628 (10 081)
Patients with diagnosis of COPD and no record of asthma (%)	2967 (1.42)
Patients with COPD with spirometry recorded—%	1999 (67.4)
Patients with COPD with FEV_1_≥50% predicted in past year	839 (28.3%)
Patients with COPD with: Diagnosis of COPD (no record asthma) + no record FEV_1_<50% predicted in past year + taking higher dosage ICS*	392 (13.2%)

*>400 μg/day beclometasone dipropionate or equivalent.

COPD, chronic obstructive pulmonary disease; ICS, inhaled corticosteroids.

### Baseline characteristics of participants

There were no significant differences in the baseline characteristics of the participants allocated to the ICS withdrawal and usual care arms (table 2). All participants were using a combination of inhaled LABA, LAMA and ICS therapies on entry to the trial. No bronchodilator inhalers had been used for 12 hours before the baseline assessment.

**Table 2:** Characteristics at baseline of all participants, participants allocated to withdrawal from ICS* and those allocated to usual care, and outcome of comparisons (Mann-Whitney U test or t-test or χ^2^ test) between withdrawal and usual care groups

	All participants, n=40	ICS withdrawal,* n=20	Usual care, n=20	P value
Age (years): mean (SD)**†**	70.10 (±9.22)	71.07 (±8.33)	69.19 (±10.16)	0.53
Male sex, n (%)‡	20 (50)	10 (50)	10 (50)	1.00
BMI (kg/m^2^): mean (SD)**†**	26.40 (±5.29)	26.09 (±5.55)	26.72 (±5.15)	0.71
Current smoker, n (%)‡	14 (35)	6 (30)	8 (40)	0.44
Tobacco exposure (pack-years): mean (SD)**†**	33.47 (±20.79)	29.83 (±17.96)	37.11 (±23.20)	0.29
AECOPD in prior year: mean (SD)**†**	0.48 (±0.51)	0.50 (±0.51)	0.45 (±0.51)	0.76
History of atopy, n (%)**‡**	65	75	55	0.18
Pre-bronchodilator FEV_1_ (L): mean (SD)†	1.74 (±0.54)	1.68 (±0.46)	1.80 (±0.49)	0.52
Post-bronchodilator FEV_1_ (L): mean (SD)†	1.82 (±0.54)	1.77 (±0.47)	1.87 (±0.61)	0.58
Pre-bronchodilator FEV_1_ % predicted: mean (SD)†	69.63 (±14.03)	69.20 (±14.41)	70.05 (±13.99)	0.85
Post-bronchodilator FEV_1_ % predicted: mean (SD)**†**	72.85 (±13.56)	73.53 (±14.12)	72.79 (±13.70)	0.98
Pre-bronchodilator FVC (L): mean (SD)**†**	2.84 (±0.79)	2.68 (±0.67)	3.00 (±0.88)	0.19
Post-bronchodilator FVC (L): mean (SD)†	2.87 (±0.78)	2.72 (±0.68)	3.03 (±0.87)	0.23
Pre-bronchodilator FEV_1_/FVC: mean (SD)**†**	0.61 (±0.09)	0.63 (±0.09)	0.60 (±0.10)	0.36
Post-bronchodilator FEV_1_/FVC: mean (SD)**†**	0.63 (±0.07)	0.65 (±0.05)	0.62 (±0.08)	0.23
Currently on LABA+LAMA+ ICS (%)‡	100	100	100	1.00
CAT score: mean (SD)**†**	15.76 (±7.54)	17.84 (±6.87)	13.68 (±7.79)	0.08
CRQ dyspnoea score: median (IQR)**§**	5.33 (4.25–6.48)	5.25 (4.60–6.50)	5.60 (4.00–6.35)	0.87
CRQ fatigue score: mean (SD)†	4.11 (±1.42)	4.04 (±1.15)	4.18 (±1.69)	0.67
CRQ emotional functioning score: mean (SD)**†**	4.80 (±1.25)	4.81 (±1.06)	4.79 (±1.44)	0.93
CRQ mastery score: median (IQR)§	5.63 (4.69–6.56)	5.25 (4.25–6.50)	5.75 (5.00–6.75)	0.10
HADS anxiety score: mean (SD)†	6.29 (±3.59)	6.42 (±3.32)	6.16 (±3.93)	0.79
HADS depression score: median (IQR)§	4.00 (1.50–5.50)	4.00 (1.00–6.00)	3.50 (1.75–5.75)	0.82
Blood eosinophil count ≥300 cells/µL (%)**‡**	8 (20)	2 (10)	6 (30)	0.11
Blood eosinophil count (cells/µL): median (IQR)§	200 (100–220)	200 (100–200)	200 (100–300)	0.41
FeNO concentration ≥25 ppb (%)**‡**	11 (28)	5 (25)	6 (30)	0.72
FeNO concentration (ppb): median (IQR)**§**	15 (9–25)	14 (8–20)	17 (12–26)	0.42
Blood periostin concentration (ng/mL): median (IQR)**§**	27.45 (19.99–60.00)	29.33 (22.53–60.00)	26.06 (16.14–60.00)	0.69

*Inhaled corticosteroids.

†t-test.

‡χ^2^ test.

§Mann-Whitney U test.

AECOPD, moderate exacerbation of COPD (max of 1 for inclusion in trial); AECOPD, acute moderate exacerbations of chronic obstructive pulmonary disease; BMI, body mass index; CAT score, Chronic Obstructive Pulmonary Disease Assessment Test; CRQ dyspnoea, Chronic Respiratory Disease Questionnaire Self-Administered Standardised Dyspnoea score; FeNO, fractional exhaled nitric oxide; FEV1, post-bronchodilatation forced expiratory volume in 1 s; FVC, post-bronchodilatation forced expiratory volume; HADS, Hospital Anxiety and Depression Scale; LABA, long-acting beta-agonis; LAMA, long-acting muscarinic antagonist.

### Acceptability of the trial design, randomisation and measurements undertaken

The processes of randomisation to withdrawal of ICS therapy or usual care, and the withdrawal of ICS therapy itself were acceptable to participants (table 3). All participants either agreed or strongly agreed that the process of randomisation to ICS withdrawal or usual care was acceptable, as were the tests they undertook during each assessment. The instructions on ICS withdrawal (for participants in the withdrawal arm), and follow-up telephone calls during ICS withdrawal were found helpful. Of patients withdrawn from ICS therapy, similar numbers reported feeling ‘better’ or ‘worse’. These responses were collected at the 6-month assessment.

**Table 3:** Acceptability of randomisation and of withdrawal of ICS therapy among participants

Acceptability assessment questions	Strongly agree	Agree	Disagree	Strongly disagree	Not available
**All participants**					
I was pleased to have a COPD check as part of the trial (n=37)	30 (81%)	7 (19%)	0	0	0
Allocation at random at the start of the trial to continue or to stop my steroid inhaler was acceptable to me (n=38)	29 (76%)	9 (24%)	0	0	0
I found the tests (blood test, breathing test, questionnaires) at each appointment acceptable (n=38)	28 (74%)	10 (26%)	0	0	0
Taking part in this research took up too much of my time (n=38)	0	0	0	24 (63%)	14 (37%)
**ICS withdrawal participants only**					
I understand the reasons why this trial is being done (n=19)	9 (47%)	10 (53%)	0	0	0
The research team answered my questions satisfactorily (n=19)	10 (53%)	9 (47%)	0	0	0
The telephone calls from the research team to check how I found the withdrawal of my inhaler were helpful (n=19)	12 (63%)	7 (37%)	0	0	0
The instructions on how to reduce the dose of my steroid inhaler were clear (n=19)	10 (53%)	9 (47%)	0	0	0
Stopping my steroid inhaler has made me feel better (n=19)	3 (16%)	6 (32%)	6 (32%)	3 (16%)	1 (4%)
Stopping my steroid inhaler has made me feel worse (n=19)	2 (11%)	5 (26%)	11 (58%)	1 (5%)	0

COPD, chronic obstructive pulmonary disease; ICS, inhaled corticosteroids.

### Measurements at baseline and at 3 and 6 months

Thirty-eight participants were followed-up at 3 and 6 months (figure 1). Four participants in the ICS withdrawal group resumed ICS therapy use after their 3-month review, of whom three resumed ICS therapy on medical advice following reported deterioration of symptoms and reductions of 15–36% in their FEV_1_ measurements, and one asked to restart despite no change in FEV_1_ or other measures. No significant differences between the ICS withdrawal group and the ICS continuation group were seen in any measures at 3-month or 6-month reviews (table 4). There was no evidence of reduction in FEV_1_, quality of life, breathlessness measures, blood eosinophil count, FeNO or blood periostin concentration at 3 or 6 months after withdrawal of ICS therapy by intention-to-treat analyses. There was no difference in rate of moderate exacerbations between the ICS withdrawal and continuation groups. No severe exacerbations were recorded. Following ICS withdrawal, an eosinophilic COPD subgroup did not emerge from the cohort. There was no evidence of severe adverse events related to withdrawal of ICS therapy.

**Table 4:** Comparison of withdrawal and usual care groups at 3 and 6 months: lung function, quality of life, breathlessness, blood eosinophil count, fractional exhaled nitric oxide and blood periostin concentration (Mann-Whitney U test or t-test)

Measure	3 months			6 months		
	**Withdrawal n=19**	**Usual care n=19**	**P value**	**Withdrawal n=19**	**Usual care n=19**	**P value**
FEV_1_ % predicted: mean (SD)*	70.95 (±18.22)	73.94 (±15.13)	0.59	72.00 (±16.59)	71.63 (±12.63)	0.94
FEV_1_/FVC: mean (SD)*	0.66 (±0.09)	0.64 (±0.13)	0.72	0.68 (±0.11)	0.66 (±0.12)	0.49
AECOPD during trial: mean (SD)*	0.20 (±0.41)	0.10 (±0.31)	0.39	0.15 (±0.37)	0.05 (±0.22)	0.30
CAT score: mean (SD)*	18.90 (±7.56)	15.24 (±7.64)	0.16	17.16 (±6.78)	16.42 (±9.28)	0.78
CRQ dyspnoea score: median (IQR)**†**	5.25 (4.40–6.00)	5.80 (4.68–7.00)	0.23	5.40 (5.00–6.20)	6.00 (4.80–6.60)	0.40
CRQ fatigue score: mean (SD)*	3.67 (±1.32)	4.18 (±1.69)	0.32	4.29 (±1.28)	4.44 (±1.65)	0.76
CRQ emotional functioning score: mean (SD)*	4.81 (±1.21)	4.84 (±1.43)	0.94	5.41 (±1.04)	4.81 (±1.67)	0.20
CRQ mastery score: median (IQR)**†**	5.00 (4.00–5.50)	5.63 (4.81–6.75)	0.06	5.50 (4.00–6.00)	5.75 (4.50–6.75)	0.43
HADS anxiety score: mean (SD)*	5.74 (±3.59)	5.28 (±4.32)	0.73	5.21 (±3.66)	5.47 (±5.05)	0.86
HADS depression score: median (IQR)**†**	5.00 (1.00–5.00)	5.50 (2.00–7.00)	0.59	4.00 (1.00–5.00)	3.00 (1.00–8.00)	0.99
Blood eosinophil count (cells/µL): median (IQR)**†**	200 (110–300)	300 (200–300)	0.31	200 (200–300)	200 (190–370)	0.40
FeNO (ppb): median (IQR)**†**	17.00 (8.50–26.00)	14.00 (9.00–17.00)	0.20	19.00 (13.00–35.50)	13.00 (10.00–18.50)	0.09
Blood periostin concentration (ng/mL): median (IQR)**†**	28.43 (6.30–60.00)	35.85 (21.96–60.00)	0.27	30.28 (27.03–60.00)	34.02 (24.77–60.00)	0.92

*t-test.

†Mann-Whitney U test.

AECOPD, acute moderate exacerbations of chronic obstructive pulmonary disease; CAT, Chronic Obstructive Pulmonary Disease Assessment Test; CRQ-SAS, Chronic Respiratory Disease Questionnaire Self-Administered Standardised Dyspnoea, Fatigue, Emotional functioning, Mastery scores; FeNO, fractional exhaled nitric oxide; FEV1, forced expiratory volume in 1 s; FVC, forced vital capacity; HADS, Hospital Anxiety and Depression Scale.

Variability in FEV_1_ (>12% and >200 mL) was observed at 3 or 6 months after baseline assessment in 18 (45%) participants, 10 in the ICS withdrawal group and 8 in the usual care group. Exploratory post-hoc analysis (online supplemental table S1) identified that, at 3 months after withdrawal of ICS, those patients demonstrating significant variability of the FEV_1_ following withdrawal of ICS therapy were more likely to report a history of atopy (p=0.01) (χ^2^ test), have elevated FeNO (≥25 ppb) (p=0.04) (Mann-Whitney U test), to report an elevated symptom burden (increase of ≥+2 in CAT score) (p=0.04) (t-test) and to report a significant deterioration in quality of life (decrease of ≥0.5 in CRQ-SAS fatigue domain score) (p=0.04) (t-test). They recorded a deterioration (decrease) in the CRQ-SAS dyspnoea domain score (p=0.04) (Mann-Whitney U test), although this did not reach the threshold for the MCID. These associations were not present at 6 months (except in the case of FeNO), by which time four of the patients in the ICS withdrawal group had recommenced ICS therapy as noted above.

## Discussion

In the present study we demonstrate that algorithm-based screening of primary care records is a practical way to identify patients given a diagnosis of COPD with mild or moderate airflow limitation and sufficiently few recent exacerbations to justify a trial of withdrawal of ICS. We also established, for the purpose of future studies addressing the consequences of such withdrawal, that eligible patients who attended for assessment were generally happy to be randomised to continue with ICS therapy or have it withdrawn under supervision. There was high compliance with withdrawal and no indication of associated significant adverse effects, including psychological problems such as withdrawal anxiety, at least for the 6-month period in which they were followed. These findings are encouraging, suggesting that ‘real life’ withdrawal of inappropriate ICS therapy from these patients will be feasible, acceptable and hazard-free.

Our study also highlights two major, potential pitfalls with this process that can be avoided with suitable anticipation and planning. The first pitfall relates to the accuracy of the records: considerable proportions of the screened patients, when reviewed in person, had more severe airways obstruction (17%) and/or a higher incidence of COPD exacerbation (22%) than documented in their records, presumably because of inevitable delay in updating. The second pitfall related to the high incidence (almost 50%) in these patients of variability of the FEV_1_ consistent with a possible diagnosis of asthma, regardless of whether they continued or discontinued existing ICS therapy, which if verified would preclude withdrawal of ICS therapy according to major guidelines such as those of the ERS^13^ and the GINA.^27^ Again, this variability of FEV_1_, either spontaneous or in response to bronchodilators, had not previously been documented in these patients’ records, while all were required to have no recorded history of asthma in order to be eligible for selection by the screening algorithm. In patients with FEV_1_ variability, the possibility of asthma was reinforced, although from an unpowered, exploratory analysis, by a significant association with history of atopy, elevated FeNO in the exhaled breath and deterioration of symptoms and quality of life after discontinuing ICS therapy. In such patients the suspicion of asthma should be formally explored. These are important considerations when estimating the numbers of patients to be screened, and the protocol used for screening, when identifying patients with true mild/moderate COPD for future clinical trials. In the current trial, these lower-than-expected numbers of recruitable patients reduced the precision of the estimates of biomarkers and outcome measures.

The clearest indication for ICS therapy in patients with COPD is reduction of exacerbation risk. In the absence of epidemiological or trial evidence of a causal relationship between ICS therapy and reduction of exacerbation risk in patients with COPD with mild or moderate airflow limitation, our feasibility data suggest that the co-primary outcomes in future trials of withdrawal of ICS therapy from these patients should be based on a combination of FEV_1_ and respiratory specific quality of life. Our data also provide evidence on which sample size estimates can be made for these primary outcomes. Our confidence in the analysis of association carried out in this trial is limited by the low number of participants.

Practically, and paradoxically, current guidelines may confound the decision to withdraw ICS therapy from those mild/moderate patients with confirmed COPD in whom the benefit/risk ratio of continued therapy is likely to be unfavourable. For example, the GOLD report recommends commencement or withdrawal of ICS treatment for these patients according to whether they have evidence of ‘≥2 moderate exacerbations or ≥1 severe exacerbation in a year’.^29^ It is readily apparent that this could easily result in the prescription of ICS therapy either continuously or alternating on and off annually. This situation is further exacerbated by the arbitrary definition of an ‘exacerbation’ in contemporary international guidelines.^28^ A moderate exacerbation, for example, is defined as an ad hoc decision by the attending physician to prescribe antibiotics or oral corticosteroids.

The finding of previously undetected airflow variability in roughly half of the patients identified from their records as suffering from mild/moderate COPD represents a significant challenge to primary care clinicians when attempting to withdraw ICS therapy from these patients. Participants with mild asthma and near normal lung function at the time of testing may exhibit ‘irreversible’ airways obstruction on some occasions, as noted in the GINA guidelines.^27^ The accuracy of ‘one off’ testing of bronchodilator reversibility for the diagnosis of asthma in patients with borderline airways obstruction is limited.^41 42^ Ideally, confirmation of asthma should come from evidence of clear, sustained peak flow variability or a positive methacholine (PC20) challenge.^43^ Our data provide preliminary evidence that, in the absence of availability of bronchial hyper-responsiveness testing in primary care, regular monitoring of lung function and symptom burden by the patient and their physician, possibly supplemented by FeNO measurement in the months following ICS withdrawal may be a useful adjunct in identifying those patients who may benefit from ICS therapy. If the diagnosis of asthma is rejected, the potential role of ICS therapy reverts to being primarily governed by the history of COPD exacerbations.^24^

Consistent with our present findings, undiagnosed asthma in many COPD clinical trials and observational studies is suggested by the presence of bronchodilator reversibility in their participants.^44 45^ Bronchodilator reversibility was found in 42% of participants in the ISOLDE trial,^45^ 54% in the UPLIFT trial^46^ and 24% in the ECLIPSE observational study.^47^ It was most prevalent in patients with GOLD stage II disease. In a cross-sectional study in primary care, 10% of patients with COPD had evidence of reversibility of airflow limitation.^44^ Reversibility of airflow limitation with a confirmed clinical diagnosis of asthma is an indication for ICS as a first-line treatment.^27^

To assess the effect of ICS withdrawal for safety purposes, comparisons were made between the ICS withdrawal and continuation groups at 3 and 6 months. Caution should be applied in the interpretation of these comparisons as, owing to its small sample size, the trial did not have the power to detect changes.

## Conclusion

This feasibility trial has demonstrated the practicability and acceptability of the withdrawal of ICS therapy from patients with mild or moderate COPD in primary care. Without withdrawal at this early stage of the disease, these patients face lifelong treatment with ICS with dubious evidence of benefit. Identification of suitable patients was difficult and laborious due to the inaccuracy of current recording of lung function and of exacerbations in the previous year. The unexpected prevalence in nearly 50% of participants of reversibility in airflow limitation suggests follow-up is advisable for at least 6 months with PC20 challenge where asthma is suspected. This feasibility trial will support recruitment planning and powering of studies of ICS withdrawal in patients diagnosed with mild or moderate COPD in primary care.

## Data Availability

Data are available upon reasonable request.
