# Supplementary material for: Withdrawal of inhaled corticosteroids from patients with COPD with mild or moderate airflow limitation in primary care: a feasibility randomised trial
Source: BMJ Open Respir Res. 2022 Aug 30;9(1):e001311. doi: 10.1136/bmjresp-2022-001311 (PMC9438092; doi:10.1136/bmjresp-2022-001311)
Supplement: Supplementary data [file bmjresp-2022-001311supp001.pdf]

Figure S1. General practice search algorithm

Search 1:

COPD, undifferentiated spirometry, age >45, BMI <35, Exclude bronchiectasis/asthma/alveolitis

Search 2:

COPD (as above) with Hx of any FEV_1 at any point

Search 3:

COPD (as above) with Hx of any FEV_1 and height

Search 4:

COPD (as above) with Hx of FEV_1% predicted ≥50 at any point

Search 5:

COPD (as above) with Hx of FEV_1% predicted ≥50 in past year

Search 6:

COPD, undifferentiated spirometry, any ICS issued in past at any point

Search 7:

COPD, undifferentiated spirometry, any ICS issued at least once in past 4 months

Search 8:

COPD, undifferentiated spirometry, high dosage ICS issued at any point in the past

Search 9:

COPD, undifferentiated spirometry, high dosage ICS issued at least once in past 4 months

Search 10:

COPD with Hx of FEV_1% predicted ≥50 any time in past, high dosage ICS issued at least once in past 4 months

Search 11:

COPD, exclude Hx of FEV_1% predicted <50 at any time in past, high dosage ICS issued at least once in past 4 months

Search 12:

COPD with Hx of FEV_1% predicted ≥50 within past year, high dosage ICS issued at least once in past 4 months

Search 13:

COPD, exclude Hx of FEV_1% predicted <50 within past year, high dosage ICS issued at least once in past 4 months

Harries TH, *et al. BMJ Open Resp Res* 2022; 9:e001311. doi: 10.1136/bmjresp-2022-001311

Figure S2. Inhaled corticosteroid therapy step-down guide

COPD Step-Down Inhaler Guide

This guide will be used by the SWAP study team to review patients diagnosed with COPD with a percentage of predicted FEV_1 of >50% with less than 2 exacerbations per year. Exclusions to step down will apply e.g. those with mixed COPD and asthma. Step down should occur no more frequently than every 2 weeks after telephone review. Patients who have been stepped down will be followed up at 2, 4 and 6 weeks after step down as a support for patients and to ensure the process is implemented. We are grateful to the Lambeth and Southwark Integrated Care Team on whose Step-down Inhaler Guide this guidance was based.

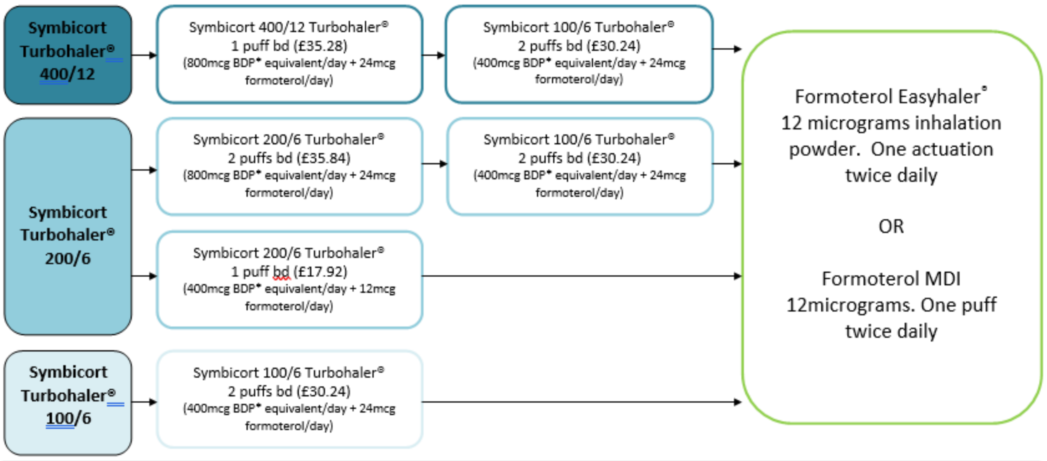

The proposed gradual reduction of steroid dose in mild and moderate COPD with <2 exacerbations per year excludes those with mixed COPD and asthma. This step down document will be used as a guide and step down will be individualised for each patient. It is important to ensure the dose of long-acting bronchodilator is maintained and not stepped down at the same time. Costs are listed as 28 day cost without spacer (BNF March 2016). *total daily dose inhaled corticosteroid in terms of beclometasone dipropionate (BDP) equivalent (standard particle size). This regime may also be used for eligible patients using Fostair®, DuoResp Spiromax (160/4.5 & 320/9) ® or using inhaled corticosteroids prescribed in a stand-alone formula.

Safe withdrawal of inhaled steroids in mild or moderate COPD (SWAP). REC Step-Down Inhaler Guide-IRAS 172251 Version 1, 24 May 2016

COPD Step-Down Inhaler Guide

This guide will be used by the SWAP study team to review patients diagnosed with COPD with a percentage of predicted FEV_1 of >50% with less than 2 exacerbations per year. Exclusions to step down will apply e.g. those with mixed COPD and asthma. Step down should occur no more frequently than every 2 weeks after a telephone review. Patients who have been stepped down will be followed up 2, 4 and 6 weeks after step down as a support for patients and to ensure the process is implemented.

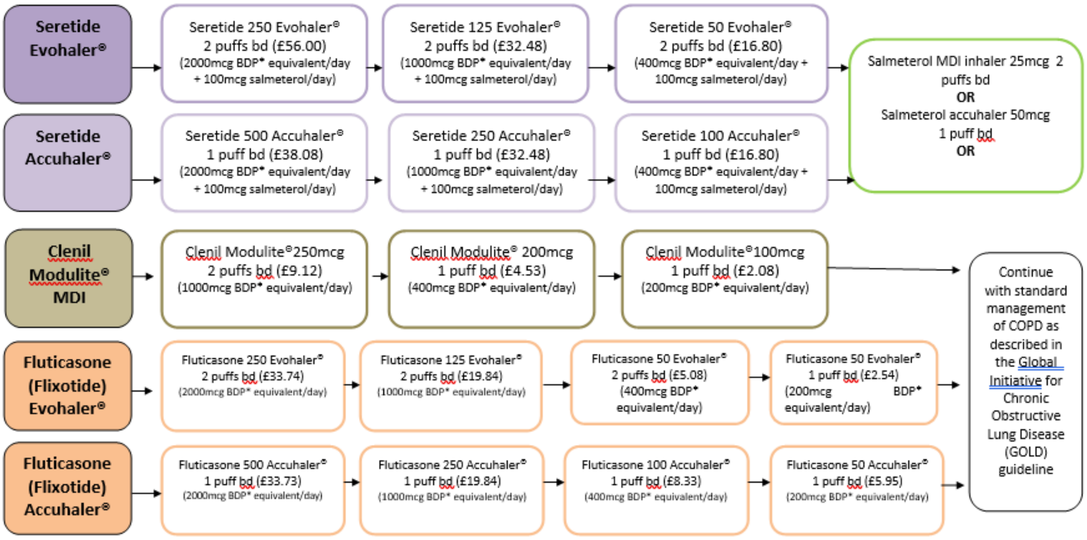

The proposed gradual reduction of steroid dose in mild and moderate COPD with <2 exacerbations per year excludes those with mixed COPD and asthma. This step down document should be used as a guide and step down individualised for each patient. It is important to ensure the dose of long acting bronchodilator is maintained and not stepped down at the same time. Costs are listed as 28 day cost without spacer (BNF March 2016). *total daily dose inhaled corticosteroid in terms of beclometasone dipropionate (BDP) equivalent (standard particle size). This regime may also be used for eligible patients using Relvar Ellipta® or using inhaled corticosteroids prescribed in a stand-alone formula.

Harries TH, *et al. BMJ Open Resp Res* 2022; 9:e001311. doi: 10.1136/bmjresp-2022-001311

Table S1. Comparison at recruitment and at 3 months between those patients with FEV_1 variability (>12% and >200ml) exhibited following ICS withdrawal and those patients who continued usual care or who had no FEV_1 variability after ICS withdrawal (Mann-Whitney U or t-test or Chi-squared test)

		FEV_1 variability after ICS withdrawal n = 10	Usual care or no FEV_1 variability after ICS withdrawal n = 28	p-value
Age (years): mean (SD)*		71 (±8.11)	70 (±9.69)	0.71
Male sex, n (%)†		5 (50%)	15 (54%)	0.85
BMI (kg/m^2): mean (SD)*		27.78 (±5.79)	26.17 (±4.87)	0.44
Tobacco Exposure (Pack Years): mean (SD)*		27.88 (±20.65)	34.92 (±20.99)	0.37
AECOPD in prior year: mean (SD)*		0.40 (±0.52)	0.54 (±0.51)	0.48
History of Atopy, n (%)†		10 (100%)	16 (57%)	**0.01**
FEV_1 (L): mean (SD)*	Baseline	1.81 (±0.35)	1.88 (±0.59)	0.69
	3 months	1.66 (±0.44)	1.88 (±0.62)	0.24
FEV_1 % predicted: mean (SD)*	Baseline	72.00 (±11.32)	73.57 (±14.66)	0.73
	3 months	67.30 (±17.40)	74.30 (±16.25)	0.29
FeNO (ppb): median (IQR)**	Baseline	11 (6-35)	15 (11-25)	0.69
	3 months	24 (8-97)	14 (9-18)	**0.04**
CAT score: mean (SD)*	Baseline	18.40 (±7.71)	14.82 (±7.39)	0.22
	3 months	21.50 (±6.88)	15.50 (±7.48)	**0.04**
Blood eosinophil count (cells/µL): median (IQR)**	Baseline	200 (100-290)	200 (100-200)	0.78
	3 months	230 (160-350)	210 (180-300)	0.91
CRQ dyspnoea score: median (IQR)**	Baseline	4.90 (4.19-5.70)	5.68 (4.19-6.50)	0.45
	3 months	4.55 (4.20-5.64)	5.67 (4.80-7.00)	**0.04**
CRQ fatigue score: mean (SD)*	Baseline	3.83 (±1.09)	4.21 (±1.53)	0.40
	3 months	3.18 (±1.09)	4.19 (±1.56)	**0.04**
CRQ emotional functioning score: mean (SD)*	Baseline	4.70 (±1.02)	4.84 (±1.33)	0.74
	3 months	4.67 (±1.34)	4.89 (±1.31)	0.67
CRQ mastery score: median (IQR)**	Baseline	5.00 (3.63-6.50)	5.75 (4.81-6.75)	0.27
	3 months	4.75 (3.06-5.56)	5.50 (4.25-6.50)	0.11
HADS anxiety score: mean (SD)*	Baseline	7.20 (±3.01)	5.96 (±3.78)	0.31
	3 months	6.20 (±3.43)	5.26 (±4.11)	0.49
HADS depression score: median (IQR)**	Baseline	4.50 (1.00-6.25)	4.00 (2.00-5.00)	0.62
	3 months	5.00 (2.00-6.00)	6.00 (2.00-7.00)	0.43
Periostin concentration (ng/ml): median (IQR)**	Baseline	39.38 (15.56-60.00)	25.82 (18.72-55.75)	0.36
	3 months	32.70 (5.98-60.00)	28.43 (18.17-60.00)	0.82

*t-test; ** Mann-Whitney U-test, †Chi-squared test. BMI: Body Mass Index. CAT score: COPD Assessment Test. FEV_1: Forced Expiratory Volume 1 second. AECOPD: acute exacerbations in COPD. FeNO: Fractional exhaled Nitric Oxide. CRQ dyspnoea: Chronic Respiratory Disease Questionnaire Self-Administered Standardized Dyspnoea score. HADS: Hospital Anxiety and Depression Scale.

Harries TH, *et al. BMJ Open Resp Res* 2022; 9:e001311. doi: 10.1136/bmjresp-2022-001311
